# Maternal behavior in transgenic mice with reduced fibroblast growth factor receptor function in gonadotropin-releasing hormone neurons

**DOI:** 10.1186/1744-9081-8-47

**Published:** 2012-09-05

**Authors:** Leah R Brooks, Carter Duyet V Le, Wilson C Chung, Pei-San Tsai

**Affiliations:** 1 University of Colorado, Integrative Physiology and Center for Neuroscience, UCB 354, Clare Small Rm. 114, Boulder, CO 80309-0354, USA

**Keywords:** GnRH, Maternal Behavior, Fibroblast Growth Factor, Estradiol, Pup Retrieval

## Abstract

**Background:**

Fibroblast growth factors (FGFs) and their receptors (FGFRs) are necessary for the proper development of gonadotropin-releasing hormone (GnRH) neurons, which are key activators of the hypothalamo-pituitary-gonadal axis. Transgenic mice that have the targeted expression of a dominant negative FGFR (dnFGFR) in GnRH neurons (dnFGFR mice) have a 30% decrease of GnRH neurons. Additionally, only 30–40% of the pups born to the transgenic dams survive to weaning age. These data raised the possibility that FGFR defects in GnRH neurons could adversely affect maternal behavior via novel mechanisms.

**Methods:**

We first determined if defective maternal behavior in dnFGFR mothers may contribute to poor pup survival by measuring pup retrieval and a battery of maternal behaviors in primiparous control (n = 10–12) and dnFGFR (n = 13–14) mothers. Other endocrine correlates of maternal behaviors, including plasma estradiol levels and hypothalamic pro-oxyphysin and GnRH transcript levels were also determined using enzyme-linked immunoassay and quantitative reverse transcription polymerase chain reaction, respectively.

**Results:**

Maternal behaviors (% time crouching with pups, time off pups but not feeding, time feeding, and total number of nesting bouts) were not significantly different in dnFGFR mice. However, dnFGFR dams were more likely to leave their pups scattered and took significantly longer to retrieve each pup compared to control dams. Further, dnFGFR mothers had significantly lower GnRH transcripts and circulating E2, but normal pro-oxyphysin transcript levels.

**Conclusions:**

Overall, this study suggests a complex scenario in which a GnRH system compromised by reduced FGF signaling leads to not only suboptimal reproductive physiology, but also suboptimal maternal behavior.

## Background

Maternal care is an essential component of offspring survival in all mammals. In mice, pups are born hairless, blind and incapable of regulating body temperature [1]. Without adequate nourishment and protection from the mother, these newborn pups would die. The critical components of maternal care in mice include lactation and behaviors such as nesting, pup retrieval, and crouching over the pups [2,3].

Neurons that synthesize gonadotropin-releasing hormone (GnRH) are the primary hormonal activators of the reproductive axis in all vertebrates. Reduced GnRH leads to profound fertility deficits in humans and other animals [4-6]. However, a role of GnRH neurons in maternal behavior, which is a critical facet of reproductive success, has never been examined. Our laboratory has generated a transgenic mouse line in which the expression of a dominant-negative fibroblast growth factor receptor (dnFGFR) has been targeted to GnRH neurons using a rat GnRH promoter [7]. Because GnRH neurons require FGF signaling for proper development [7,8], these mice suffer a significant reduction in GnRH neurons after birth [7]. A serendipitous discovery is the difficulty involved in propagating the dnFGFR mice (Tsai, unpublished data). This is in part due to their documented smaller litter size and shorter reproductive lifespan [7], but may also be due to compromised maternal behavior and offspring survival. These mice are useful for examining the connection between the maternal behavior and the GnRH system since they harbor only GnRH neuron-specific defects, thereby eliminating confounding effects originating from other tissues. In a sense, this model represents a condition in which the GnRH system is significantly compromised but not completely incapacitated. Another potential mouse model with a specific loss of the GnRH system is the *hpg* mouse harboring a deletion mutation on the *GnRH* gene [5]. However, this mouse is sterile and cannot undergo pregnancy or exhibit natural maternal behavior.

This study investigates maternal behavior of dnFGFR mice, a model characterized by reduced GnRH neuron number and difficult offspring propagation. We find specific aspects of maternal behavior are in fact disrupted in dnFGFR dams. We further examined if deficits in two hormones involved in lactation and maternal behavior, oxytocin (OT) mRNA and circulating estradiol (E2) [9], could contribute to these behavioral disruptions. Overall, our studies suggest a novel scenario in which a GnRH system compromised by reduced FGF signaling can lead to not only suboptimal reproductive physiology, but also suboptimal maternal behavior.

## Methods

### Subjects

dnFGFR mice were generated by Tsai et al. (2005). These mice have the expression of a dnFGFR cassette targeted specifically to GnRH neurons using a rat GnRH promoter, thereby compromising the ability of GnRH neurons to respond to FGF signaling. As previously described by Tsai et al. (2005), dnFGFR mice were generated on a background of C57BL/6J × DBA/2JF1. These animals were then bred to homozygosity to maximize dnFGFR expression. Non-transgenic animals resulting from heterozygous crosses were used as controls to ensure the same genetic background as the transgenic mice. All animals were kept on a 12 h light (from 0800 to 2000), and 12 h dark cycle and fed water and rodent chow ad *libitum*. All animal procedures complied with the protocols approved by the Institutional Animal Care and Use Committee at the University of Colorado.

### Pup survival

To determine the breeding success of control or dnFGFR mice, we evaluated the percentage of pups born by control (n = 6) or dnFGFR (n = 22) dams that survive to weaning age. Percent pup survival on postnatal day (PD) 21 (weaning) was calculated for each dam’s first three litters. Pup survival to PD1 was calculated in a separate cohort for control (n = 4) and dnFGFR (n = 6) litters. We also investigated pup weights on the day of birth (PD0), PD1 and PD2 (n = 29 and n = 6 for pups born to control and dnFGFR dams, respectively).

### Maternal behavior and pup retrieval test

We performed a maternal behavior assay and the pup retrieval test to determine if maternal behavior is impacted by disruptions in the GnRH system. For all behavioral observations, primiparous control (n = 10–12) or dnFGFR (n = 13–14) females at 3–6 months of age were mated to control males. This mating scheme generated two offspring genotypes: heterozygous dnFGFR (by dnFGFR dams) and control pups (by control dams). Our observations showed that heterozygous dnFGFR and control pups did not survive differently under the care of the same dam. When heterozygous females were mated with control males, 53.1 ± 7.05% (n = 3 dams, 9 ± 2.5 pups/litter) of the offspring that survived to weaning age were heterozygous dnFGFR pups, with the remainder being control pups, a ratio consistent with Mendelian genetics. These data suggest differences in pup survival would likely come from the dams, not the pups themselves. The pregnant females were singly housed 3–10 days prior to parturition. The maternal behavior assay and pup retrieval test were performed between the hours 2100 and 2300, and given the high mortality rate of pups born by dnFGFR mothers (see Results section), were only performed within 24 hours of birth (PD0). Multiple days of testing were not possible due to the early mortality of some pups. During the maternal behavioral assay, the dam with her pups were videotaped undisturbed in the dam’s home cage for an hour. The cage was illuminated by one 40-W red light bulb suspended above the cage. These videotapes were analyzed by an observer blind to the identity of the animals and scored for the following parameters: percent time crouching over pups (nursing/crouching/resting), percent time off nest (not in contact with pups), percent time feeding, total number of nesting bouts (moving nest material towards nest or rearranging the current nest material) and the location of the pups in relation to the nest immediately before and during testing.

Immediately after the maternal behavior assay, the pup retrieval test was conducted for a total duration of 15 minutes. In this test, the dam was removed from the cage and three of her pups were placed in three different corners [10]. The remaining pups were counted and put in a separate cage. The dam was then returned to the center of the home cage and the latencies to sniff one of the pups, retrieve each of the three pups, and crouch over them for longer than a minute were recorded. The total duration of crouching over the pups and the number of nesting bouts were also scored. If a dam failed to complete the retrieval in 15 minutes, a score of 900 s was assigned. Following the retrieval test, the pups were weighed and the presence of milk in the pups’ stomachs was recorded. Dams were subsequently sacrificed by decapitation and total RNA from their brains was isolated for quantitative reverse transcription polymerase chain reaction (qPCR). Trunk blood was also collected for an E2 enzyme immunoassay (EIA).

### Reverse transcription quantitative polymerase chain reaction (qPCR)

In addition to adequate maternal care, pup survival also depends on milk production and ejection, the latter of which requires the presence of OT. As a control to verify that milk was available to pups born to dnFGFR dams, we examined the presence of milk in the pups’ stomach and quantified the levels of pro-oxyphysin transcript in the hypothalamus by qPCR. Because dnFGFR mice have fewer GnRH neurons, we also performed GnRH qPCR to determine whether GnRH transcript was reduced. Following the retrieval test, dams were sacrificed by decapitation and the brains dissected and blocked (n = 6 control and n = 7 dnFGFR dams). The preoptic area (POA) represents the brain region between the posterior borders of olfactory bulbs and the optic chiasm. The hypothalamus (HYPO) represents the brain region between the optic chiasm and caudal mammillary body. These tissue blocks were immediately placed on dry ice and kept frozen at −70°C until processing. Total RNA was isolated with TRIzol (Invitrogen, Carlsbad, CA) according to the manufacturer’s instructions. One μg of total RNA was used for the synthesis of cDNA using the Superscript III First-Strand cDNA Synthesis kit (Invitrogen). Real-time qPCR of GnRH and pro-oxyphysin transcripts was performed using the Fast Start DNA Master SYBR Green I kit (Roche Pharmaceuticals, Indianapolis, IN), as previously described [11]. Absolute quantification was performed using vectors containing the full-length mouse GnRH or pro-oxyphysin cDNA as standards. Primer sequences were: 5′CTGCTGACTGTGTGTTTGGAAGG (forward) and 5′CCTGGCTTCCTCTTCAATCA (reverse) for GnRH, and 5′-CAGGGCGAAGGCAGGTAGTT (forward) and 5′-GTCTCGCTTGCTGCCTGCTT (reverse) for pro-oxyphysin. All amplifications were performed for 40 cycles with annealing temperature of 60°C. Following data acquisition, data from the POA and HYPO blocks were combined to determine the total hypothalamic content of pro-oxyphysin or GnRH mRNA. These data were presented as “hypothalamic” in the Results section.

### E2 enzyme linked immunoassay (EIA)

To determine whether disruptions in the GnRH system impact circulating E2, we performed E2 EIA on plasma samples. Following the pup retrieval test, dams were sacrificed and trunk blood collected into heparinized tubes for plasma isolation (n = 4 control and n = 6 dnFGFR dams). Plasma E2 was measured using a commercial EIA kit validated for the mouse (Cayman Chemical Company, Ann Arbor, MI) according to manufacturer’s instructions. Two serially diluted doses were measured for each sample using a BioTek Synergy^TM^ HT plate reader and Gen5 software. Samples with serial dilutions not parallel to the standard were excluded from the analysis. The intra- and inter-assay coefficients of variation were 13% and 8.2%, respectively, and the limit of detection was 19 pg/ml.

### Data analysis

Fisher’s exact test was used to analyze the differences in the percentages of dams with scattered pups and dams with pups scattered that were also non-retrievers. Chi-square analysis was used to analyze pups with milk in their stomachs. Friedman’s test was used to determine whether pup survival to weaning improved with successive litters within each genotype. Two-way repeated measures ANOVA followed by the Holm-Sidak multiple comparison test was used to analyze pup weights and the latency to retrieve pups. All other data were analyzed using the Student’s *t*-test or the Mann–Whitney Rank Sum test. SigmaPlot v.11 (Systat Software Inc.) was used for all statistical analyses. If subjects were two standard deviations from the mean they were considered statistical outliers for that test and were removed from that analysis. Data were considered significant when p < 0.05.

## Results

### Pup survival

**Figure 1:**
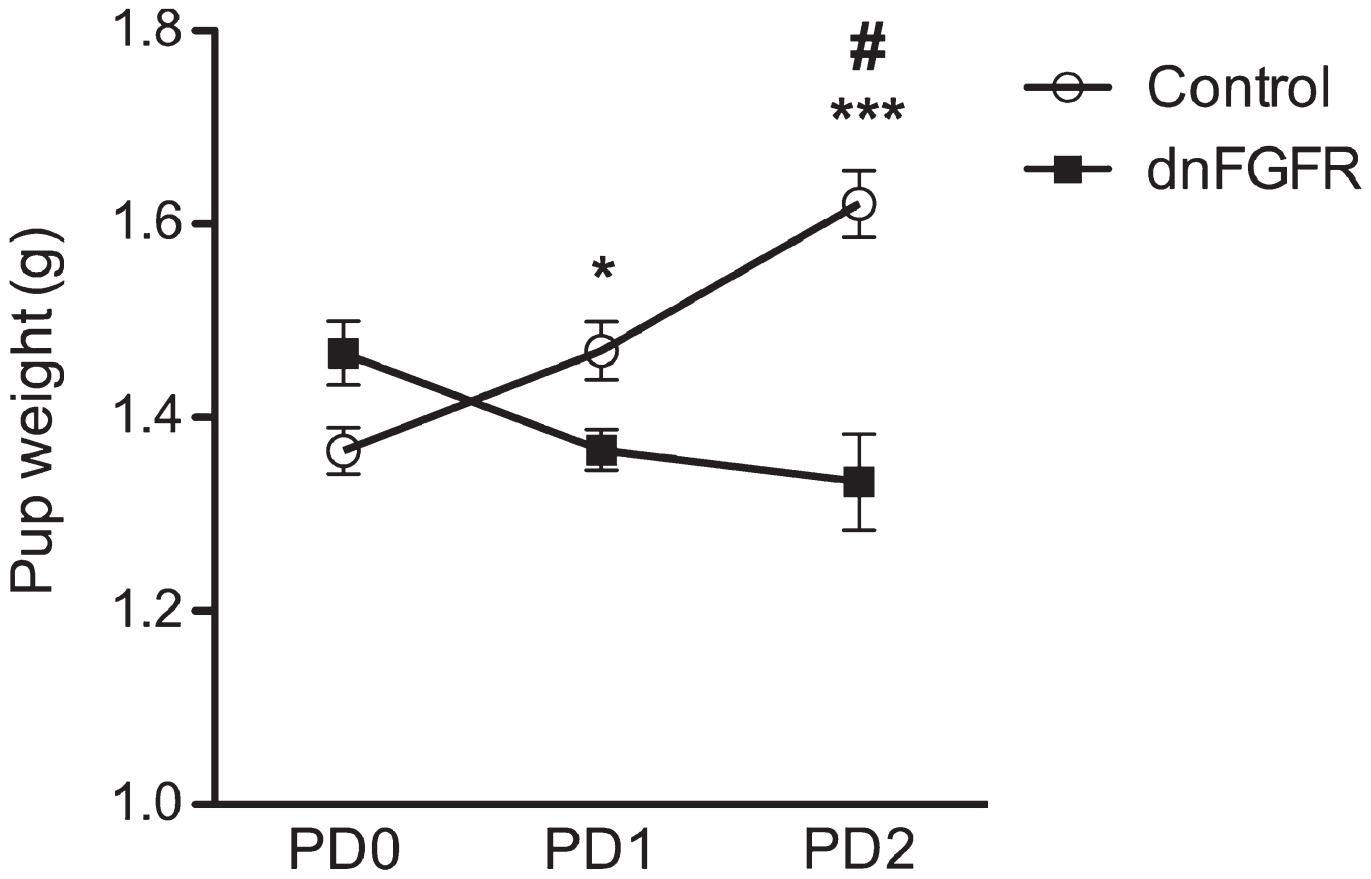
**Pup weights from PD0 to PD2. Pups born to control but not dnFGFR dams gain weight from PD0 to PD2. **On PD2, pups born by dnFGFR dams weigh significantly less than pups born to control dams. Control n = 29 and dnFGFR n = 6 pups. Bars represent the mean ± SEM. *p < 0.05 control PD0 vs. PD1; ***p < 0.001 control PD0 vs. PD2 and PD1 vs. PD2; #p < 0.001 PD2 control vs. dnFGFR.

dnFGFR dams have low pup survival compared to controls. For pups born to control dams (n = 6 dams, 8.5 ± 0.63 pups/litter), 100.0 ± 0.0, 97.2 ± 2.8, and 100.0 ± 0.0% survive to weaning age for litters 1, 2, and 3, respectively. For pups born to dnFGFR dams (n = 22 dams, 6.6 ± 0.41 pups/litter), 33.2 ± 9.1, 38 ± 9.7, and 39 ± 9.7% survive to weaning age for litters 1, 2 and 3, respectively. Pup survival does not improve or change with subsequent litters for either genotype (p > 0.05). A separate cohort of mice was analyzed for pup survival to PD1. In this cohort, 20.8 ± 13.6% of pups born to dnFGFR dams (n = 6 dams, 6.2 ± 0.87 pups/litter) survive to PD1 compared to 100% survival in pups born to control dams (n = 4 dams, 7.6 ± 1.5 pups/litter) (p = 0.01). There was a significant interaction between genotype and day (F_2,66_ = 8.33, p < 0.001) and a main effect of genotype (F_1,33_ = 5.77, p = 0.022) on pup weight (Figure 1). Holm-Sidak multiple comparison test revealed that pups born to control dams gained weight from PD0 to PD2 (Figure 1). In contrast, pups born to dnFGFR dams failed to gain weight and consequently weighed significantly less than pups born to control dams on PD2 (Figure 1; p < 0.001). There was not a main effect of day on pup weight.

### Maternal behavior and pup retrieval

**Figure 2:**
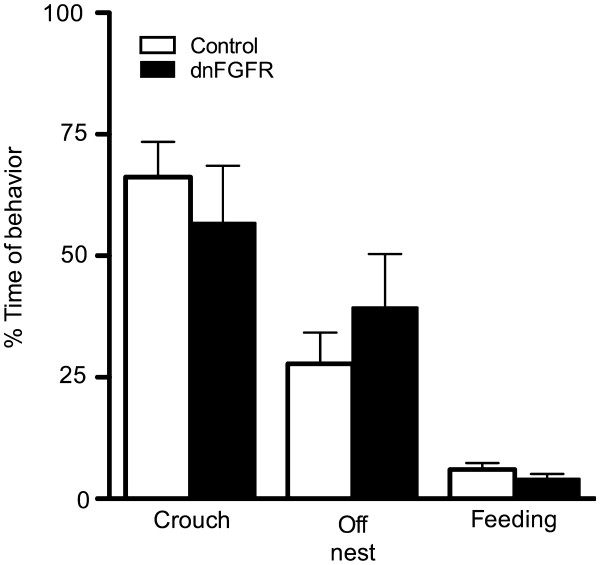
**Maternal behaviors assessed in control and dnFGFR dams. **There were no significant differences between the genotypes in percentage of time each dam spent crouching over pups, off nest, and feeding. Control n = 11 and dnFGFR n = 14 dams. Bars represent the mean ± SEM.

**Figure 3:**
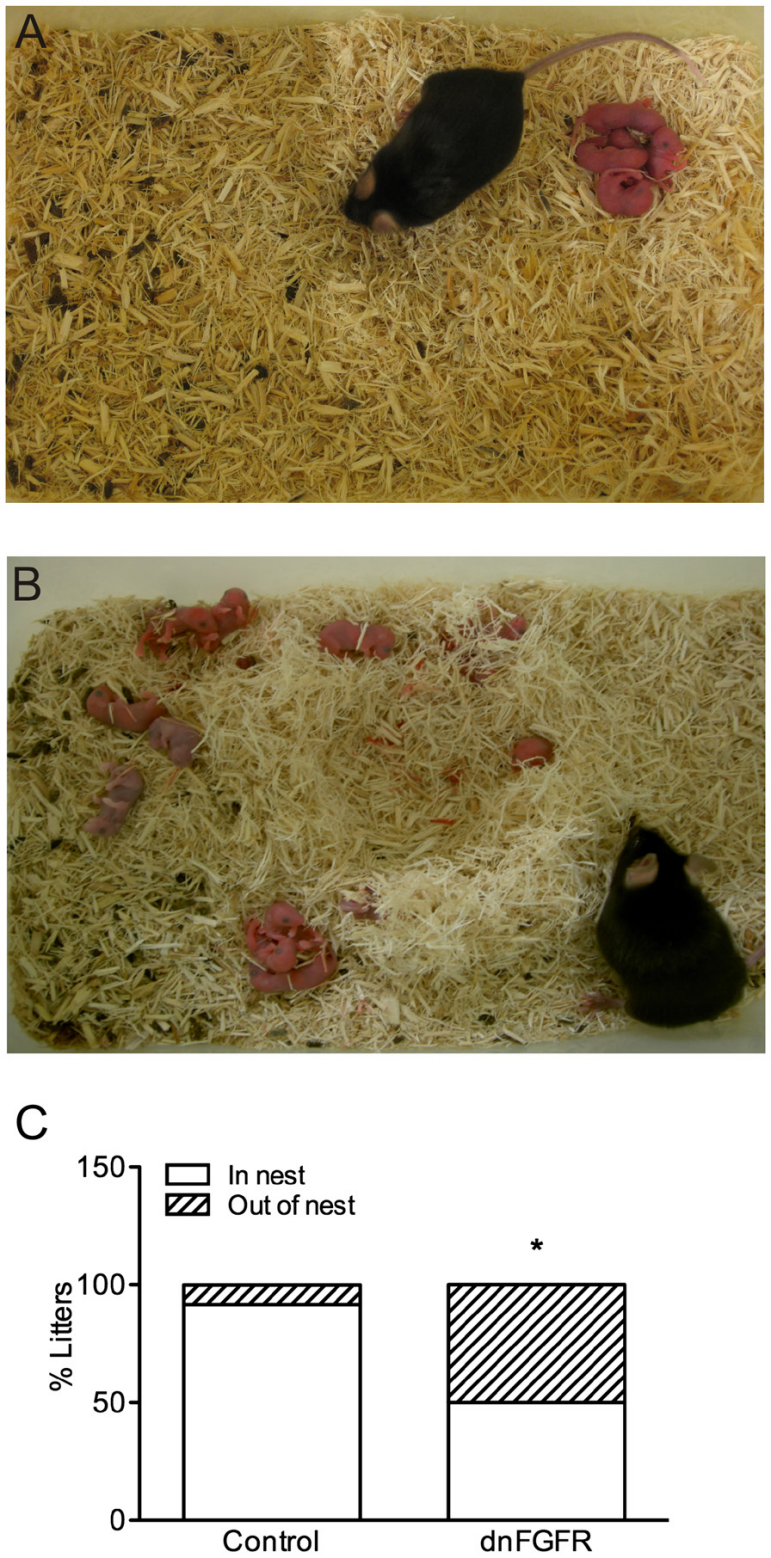
**Illustration of pup scattering in the home cage during the maternal behavior assay.** Significantly more dnFGFR dams had pups scattered around the cage during behavioral testing (B and C) than control dams (A and C). Control n = 12 and dnFGFR n = 14 dams. Bars represent percent of litters found in nest vs. out of nest for each genotype. *p < 0.05 compared to control.

Given the high mortality of pups born to dnFGFR dams by PD1 (see Results section), maternal behavior and the pup retrieval test were only performed on PD0. Maternal behavior of each dam was recorded for one hour and scored for the following parameters: time crouching over pups in the nest, time off nest but not feeding, time feeding, and total number of nesting bouts and position of pups in relation to the nest. No differences were found between maternal genotypes for any of the maternal behaviors, including time spent crouching over their pups (Figure 2), time spent off the nest (Figure 2), time spent feeding (Figure 2), and the total number of nesting bouts (4.2 ± 0.98, and 5.3 ± 0.98 for control and dnFGFR dams, respectively). However, there was a significant difference between genotypes in the location of pups relative to the nest (p = 0.036). Of the 14 dnFGFR dams, seven of them had pups scattered outside the nest (Figure 3B, C). In comparison, only one of the twelve control dams exhibited this pup-scattering phenotype (Figure 3A, C).

**Figure 4:**
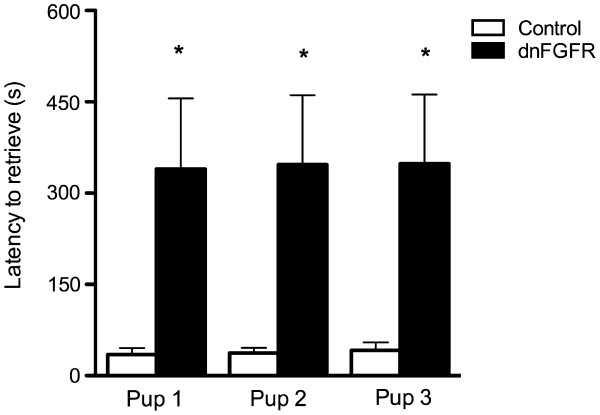
**The time required to retrieve each pup was assessed in control and dnFGFR dams during the pup retrieval test. **It took dnFGFR females significantly longer to retrieve each pup. Control n = 10 and dnFGFR n = 14 dams. Bars represent the mean ± SEM. *p < 0.05 compared to control for each pup.

Two-way repeated ANOVA indicated a significant main effect of genotype on pup retrieval (F_1, 22_ = 5.036, p = 0.035). Holm-Sidak post-hoc analysis revealed that dnFGFR females took significantly longer to retrieve each pup to the nest than control (Figure 4; p = 0.036, 0.034, 0.036 for Pup 1, 2, 3, respectively). There was no main effect of pup or a genotype x pup interaction. During the retrieval test, several behavioral parameters were also scored. There were no significant differences between genotypes in any of these retrieval test behaviors (Table 1). Interestingly, 67% of dams that failed to retrieve pups during the pup retrieval test (compared to only 6% of dams that successfully retrieved pups) scattered their pups in the maternal behavior assay (p < 0.001), suggesting dams that scattered pups were unlikely to retrieve their pups.

**Table 1:** Other behavioral assessments during the pup retrieval test

	**Latency to sniff**	**Latency to crouch**	**Crouch duration**	**Nesting bouts**
	**(s)**	**(s)**	**(s)**	**(#)**
**Genotype**	**(mean ± SEM)**	**(mean ± SEM)**	**(mean ± SEM)**	**(mean ± SEM)**
Control	3.80 ± 0.83	236.3 ± 23.06	456.3 ± 54.77	6.3 ± 1.27
dnFGFR	4.92 ± 1.30	491.14 ± 93.10	351.8 ± 90.59	4.2 ± 0.80

Summary of other behaviors during the 15-min pup retrieval test. No differences between genotypes were found for any behavior. Control n = 10 and dnFGFR n = 13–14 dams.

### qPCR of pro-oxyphysin and GnRH transcripts

**Figure 5:**
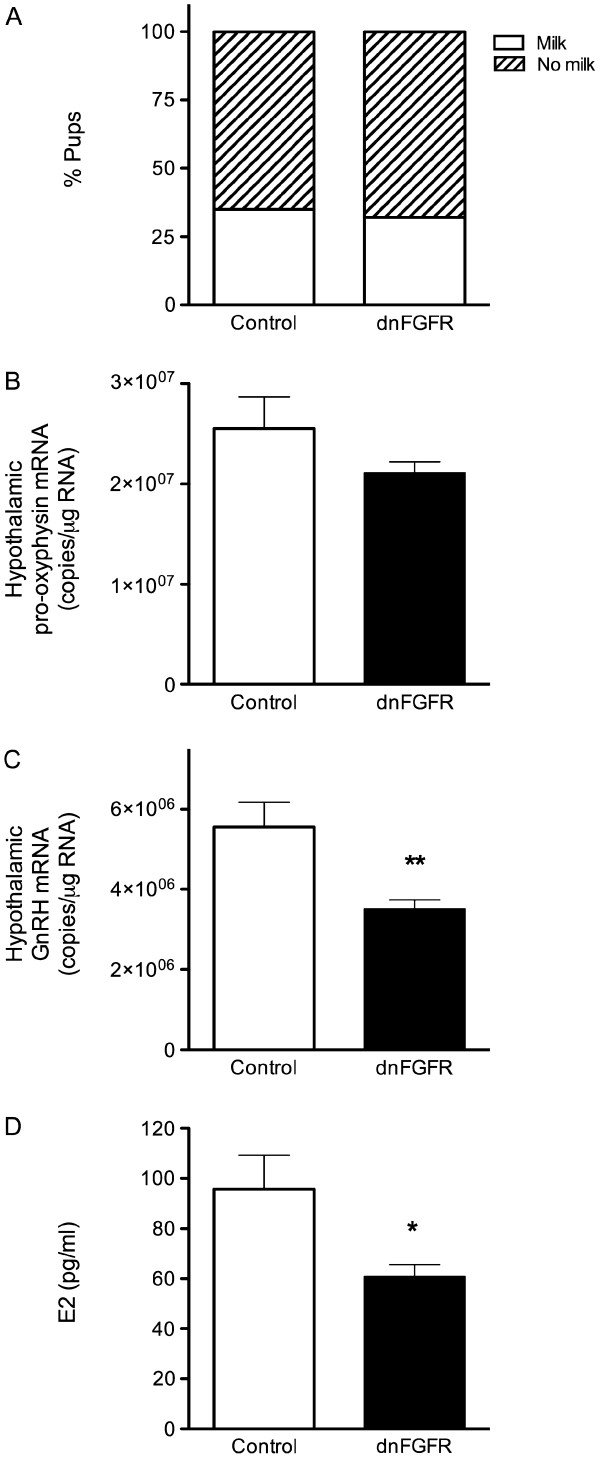
**Physiological and endocrine correlates of maternal behavior in control and dnFGFR mothers. **(**A**) Percent pups with or without milk in their stomachs that are born by control or dnFGFR dams. N = 46 and 71 for pups born to control and dnFGFR dams, respectively. (**B**, **C**) Total hypothalamic pro-oxyphysin (**B**) or GnRH (**C**) transcript in post-partum dams. Control n = 6 and dnFGFR n = 7 dams. (**D**) Plasma E2 in control and dnFGFR post-partum dams. Control n = 4 and dnFGFR n = 6 dams. Bars represent the mean ± SEM. *p < 0.05; **p < 0.01.

In addition to adequate maternal care, pup survival also depends on milk production and ejection, the latter of which depends on OT. To examine if pups received milk from the mothers, we determined if pups born to dnFGFR dams had milk in their stomachs, and if OT transcript levels were similar between control and dnFGFR dams. Because dnFGFR mice had fewer GnRH neurons [7], we also performed GnRH qPCR to determine whether GnRH transcript was also reduced. The percentages of pups with milk in their stomachs were similar between control and dnFGFR mothers (Figure 5A; p = 0.947). No difference in hypothalamic pro-oxyphysin transcript was found between the control and dnFGFR mothers (Figure 5B; p = 0.628). In contrast, there was a significant effect of genotype on the amount of hypothalamic GnRH transcript (Figure 5C; p = 0.007).

### E2 EIA

To determine whether disruptions in the GnRH system impact the levels of estradiol, we performed an E2 EIA on plasma collected from post-partum control and dnFGFR dams. dnFGFR mothers had significantly lower circulating E2 than controls (Figure 5D; p = 0.038).

## Discussion

In this study, we provide novel evidence that defects in the GnRH system may reduce reproductive success through mechanisms unrelated to reduced fertility. Namely, a GnRH system compromised by FGF signaling deficiency disrupts important pup-gathering and retrieval behaviors, thereby contributing to reduced offspring care. Although a direct causal link has not been established, this reduced care may contribute to the reduced survival of pups born to dnFGFR dams. The basis of the behavioral defect is presently unclear, but two mechanisms are possible. The first is supported by indirect evidence and posits that a disrupted GnRH system may directly and adversely affect the maternal behavior neurocircuits upon which it impinges. The second is that the disrupted maternal behavior in dnFGFR mice may be secondary to reduced circulating E2. These two possibilities are discussed below.

The first possibility is that the disruption of the GnRH system directly impacts the maternal circuits. GnRH neurons are predominantly found in the medial POA of the hypothalamus (MPA) [12-14]. This heterogeneous region rostral to the optic chiasm is central to rodent reproductive behaviors, including maternal behavior. At present, the precise neural connections linking the GnRH system and maternal behavior neurocircuits are unclear. What is clear is that a number of behavioral neurocircuits such as the dopaminergic, serotonergic, olfactory and oxytocinergic systems have all been found to play a role in maternal behaviors, and each of these systems has projections to and from the MPA [3,15-19]. Since GnRH neurons are located in the MPA and express receptors for dopamine, serotonin, and OT, it is possible that the GnRH system and the MPA neurocircuits mediating maternal behavior share a number of common input and output projections [17,20,21]. Supporting this notion, GnRH was shown to act as a neuromodulator by modifying the responses of cultured MPA neurons to norepinephrine and serotonin [22]. Further, mapping studies with a transneuronal tracer expressed specifically in GnRH neurons reported connections between GnRH neurons and numerous brain regions important for maternal behavior, including the nucleus accumbens, bed nucleus of the stria terminalis, amygdala, septum and numerous hypothalamic nuclei [23]. These regions also express type I GnRH receptor [24], which is a G protein-coupled receptor acting primarily through the G protein, G_q/11_[25]. Interestingly, forebrain G_q/11_ knockout mothers failed to retrieve pups to the nest and had no pup survive to weaning age [26]. These results paralleled our current findings and suggest a decreased activation of the GnRH receptor may adversely impact maternal care through direct or transsynaptic alterations of the maternal behavior circuits.

In addition to having reduced GnRH neuron number, transcript and peptide, dnFGFR mice also have aberrant FGF signaling in GnRH neurons [7]. Because FGF signaling activates a variety of signal transduction pathways which include phospholipase Cγ, extracellular signal-regulated kinase (ERK), and the phosphoinositide 3-kinase/Akt pathways, the consequent disruption in intracellular signaling can directly alter the biology of GnRH neurons [27]. This may render GnRH neurons less able to respond to afferents that rely upon them to convey signals to the maternal circuitries. For example, olfactory cues are important instigators for maternal behavior in mice [28-31]. Sensory afferents from the olfactory system have been shown to mediate maternal behaviors and also indirectly connect with GnRH neurons [23,32,33]. In this respect, the GnRH system may serve as an intermediary or modulator of olfactory projections to maternal circuitries in the MPA [23]. Disruption of GnRH neuronal biology as the result of FGF signaling deficiency could then lead to defects in this modulatory or connective role.

An alternate hypothesis is that a deficiency in E2 in dnFGFR mice might be the cause of maternal behavior deficits. The reduction of GnRH neurons, transcript and peptide content in dnFGFR mice may lead to reduced gonadotropins and ultimately circulating E2. Several lines of evidence support the importance of E2 in rat maternal behavior [34-36]. For example, direct infusions of E2 into the MPA elicit rat maternal behaviors [37]. Additionally, MPA maternal behavior circuits express abundant estrogen receptor alpha (ERα), implicating a direct influence of E2. The story has been less clear in mice. A study supports a direct link between maternal behaviors and estrogen signaling by demonstrating that ERα knockout (KO) virgin female mice exhibit impaired pup retrieval and increased infanticide [38]. However, other evidence suggests that pup-oriented maternal behaviors in virgin female mice are independent of estrogens [3]. Further, Stolzenberg and Rissman (2011) have shown repeated pup exposure is the most important factor in maternal behavior and that estrogens play only a facilitatory role [39]. Given the controversial role of E2 in mouse maternal behavior, we favor the hypothesis that deficient maternal behavior in dnFGFR mice is more causally linked to a compromised GnRH system than reduced E2.

We describe here an interesting phenomenon in which reduced GnRH is coupled to compromised maternal behaviors. A disrupted GnRH system thus adversely impacts not only fertility, but may also impact offspring survival. Given that hypothalamic OT mRNA and milk availability are normal in dnFGFR dams, lactational defects are unlikely the cause of poor survival in pups born to dnFGFR dams. Rather, disrupted maternal behavior that reduces pup gathering into the nest is more likely a contributor to poor pup survival. We have proposed two mechanisms that could cause this disrupted maternal behavior in dnFGFR mice: (1) suboptimal GnRH connectivity with maternal neurocircuits as a result of decreased FGF signaling and (2) reduced E2 in dnFGFR mice leading to impaired maternal neurocircuits. Regardless of the mechanism, this study establishes that a functional GnRH system guided by FGF signaling is critical for maternal behaviors and potentially offspring survival.

## Competing interests

The authors declare that they have no competing interests.

## Authors’ contributions

LRB wrote the manuscript and designed, performed, and analyzed all of the experiments. CDVL participated in conducting the behavioral experiments. WCC contributed to the design. PST participated in the design, analysis, and contributed to the manuscript. All authors read and approved the final manuscript.

## References

[B1] Crawley J What’s wrong with my mouse 2000 Wiley-Liss, New York

[B2] Shoji H Kato K Maternal behavior of primiparous females in inbred strains of mice: a detailed descriptive analysis Physiol Behav 2006 89 320 328 16842828 10.1016/j.physbeh.2006.06.012

[B3] Numan M Insel TR The neurobiology of parental behavior 2003 Springer, New York

[B4] Chan YM de Guillebon A Lang-Muritano M Plummer L Cerrato F Tsiaras S Gaspert A Lavoie HB Wu CH Crowley WF Jr GNRH1 mutations in patients with idiopathic hypogonadotropic hypogonadism Proc Natl Acad Sci USA 2009 106 11703 11708 19567835 10.1073/pnas.0903449106 PMC2710623

[B5] Mason AJ Hayflick JS Zoeller RT Young WS 3rd Phillips HS Nikolics K Seeburg PH A deletion truncating the gonadotropin-releasing hormone gene is responsible for hypogonadism in the hpg mouse Science 1986 234 1366 1371 3024317 10.1126/science.3024317

[B6] Bouligand J Ghervan C Tello JA Brailly-Tabard S Salenave S Chanson P Lombes M Millar RP Guiochon-Mantel A Young J Isolated familial hypogonadotropic hypogonadism and a GNRH1 mutation N Engl J Med 2009 360 2742 2748 19535795 10.1056/NEJMoa0900136

[B7] Tsai PS Moenter SM Postigo HR El Majdoubi M Pak TR Gill JC Paruthiyil S Werner S Weiner RI Targeted expression of a dominant-negative fibroblast growth factor (FGF) receptor in gonadotropin-releasing hormone (GnRH) neurons reduces FGF responsiveness and the size of GnRH neuronal population Mol Endocrinol 2005 19 225 236 15459253 10.1210/me.2004-0330

[B8] Dode C Levilliers J Dupont JM De Paepe A Le Du N Soussi-Yanicostas N Coimbra RS Delmaghani S Compain-Nouaille S Baverel F Loss-of-function mutations in FGFR1 cause autosomal dominant Kallmann syndrome Nat Genet 2003 33 463 465 12627230 10.1038/ng1122

[B9] Pfaff D Waters E Khan Q Zhang X Numan M Minireview: estrogen receptor-initiated mechanisms causal to mammalian reproductive behaviors Endocrinology 2011 152 1209 1217 21325045 10.1210/en.2010-1007 PMC3060638

[B10] Champagne FA Curley JP Keverne EB Bateson PP Natural variations in postpartum maternal care in inbred and outbred mice Physiol Behav 2007 91 325 334 17477940 10.1016/j.physbeh.2007.03.014

[B11] Brooks LR Chung WC Tsai PS Abnormal hypothalamic oxytocin system in fibroblast growth factor 8-deficient mice Endocrine 2010 38 174 180 21046478 10.1007/s12020-010-9366-9 PMC3093295

[B12] Renaud LP Day TA Ferguson AV CNS regulation of reproduction: peptidergic mechanisms Brain Res Bull 1984 12 181 186 6144374 10.1016/0361-9230(84)90187-4

[B13] Witkin JW Paden CM Silverman AJ The luteinizing hormone-releasing hormone (LHRH) systems in the rat brain Neuroendocrinology 1982 35 429 438 6759973 10.1159/000123419

[B14] Franklin KBJ Paxinos G The mouse brain in stereotaxic coordinates 2007 3 Academic Press, New York

[B15] Leckman JF Herman AE Maternal behavior and developmental psychopathology Biol Psychiatry 2002 51 27 43 11801229 10.1016/s0006-3223(01)01277-x

[B16] Flannelly KJ Kemble ED Blanchard DC Blanchard RJ Effects of septal-forebrain lesions on maternal aggression and maternal care Behav Neural Biol 1986 45 17 30 3954712 10.1016/s0163-1047(86)80002-4

[B17] Gammie SC Current models and future directions for understanding the neural circuitries of maternal behaviors in rodents Behav Cogn Neurosci Rev 2005 4 119 135 16251728 10.1177/1534582305281086

[B18] Calamandrei G Keverne EB Differential expression of Fos protein in the brain of female mice dependent on pup sensory cues and maternal experience Behav Neurosci 1994 108 113 120 8192837 10.1037//0735-7044.108.1.113

[B19] Lerch-Haner JK Frierson D Crawford LK Beck SG Deneris ES Serotonergic transcriptional programming determines maternal behavior and offspring survival Nat Neurosci 2008 11 1001 1003 19160496 10.1038/nn.2176 PMC2679641

[B20] Li S Pelletier G Involvement of serotonin in the regulation of GnRH gene expression in the male rat brain Neuropeptides 1995 29 21 25 7566509 10.1016/0143-4179(95)90052-7

[B21] Numan M Stolzenberg DS Medial preoptic area interactions with dopamine neural systems in the control of the onset and maintenance of maternal behavior in rats Front Neuroendocrinol 2009 30 46 64 19022278 10.1016/j.yfrne.2008.10.002

[B22] Pan JT Kow LM Pfaff DW Modulatory actions of luteinizing hormone-releasing hormone on electrical activity of preoptic neurons in brain slices Neuroscience 1988 27 623 628 3146034 10.1016/0306-4522(88)90293-x

[B23] Boehm U Zou Z Buck LB Feedback loops link odor and pheromone signaling with reproduction Cell 2005 123 683 695 16290036 10.1016/j.cell.2005.09.027

[B24] Albertson AJ Navratil A Mignot M Dufourny L Cherrington B Skinner DC Immunoreactive GnRH type I receptors in the mouse and sheep brain J Chem Neuroanat 2008 35 326 333 18439800 10.1016/j.jchemneu.2008.03.004 PMC2435296

[B25] Millar RP Lu Z-L Pawson AJ Flanagan CA Morgan K Maudsley SR Gonadotropin-Releasing Hormone Receptors Endocr Rev 2004 25 235 275 15082521 10.1210/er.2003-0002

[B26] Wettschureck N Moers A Hamalainen T Lemberger T Schutz G Offermanns S Heterotrimeric G proteins of the Gq/11 family are crucial for the induction of maternal behavior in mice Mol Cell Biol 2004 24 8048 8054 15340067 10.1128/MCB.24.18.8048-8054.2004 PMC515047

[B27] Reuss B von Bohlen und Halbach O Fibroblast growth factors and their receptors in the central nervous system Cell Tissue Res 2003 313 139 157 12845521 10.1007/s00441-003-0756-7

[B28] Belluscio L Gold GH Nemes A Axel R Mice deficient in G(olf) are anosmic Neuron 1998 20 69 81 9459443 10.1016/s0896-6273(00)80435-3

[B29] Gandelman R Zarrow MX Denenberg VH Reproductive and maternal performance in the mouse following removal of the olfactory bulbs J Reprod Fertil 1972 28 453 456 5018060 10.1530/jrf.0.0280453

[B30] Gandelman R Zarrow MX Denenberg VH Myers M Olfactory bulb removal eliminates maternal behavior in the mouse Science 1971 171 210 211 5540330 10.1126/science.171.3967.210

[B31] Zarrow MX Gandelman R Denenberg VH Lack of nest building and maternal behavior in the mouse following olfactory bulb removal Horm Behav 1971 2 227 238

[B32] Samuelsen CL Meredith M Categorization of biologically relevant chemical signals in the medial amygdala Brain Res 2009 1263 33 42 19368822 10.1016/j.brainres.2009.01.048 PMC2798152

[B33] Dulac C Torello AT Molecular detection of pheromone signals in mammals: from genes to behaviour Nat Rev Neurosci 2003 4 551 562 12838330 10.1038/nrn1140

[B34] Pedersen CA Oxytocin control of maternal behavior. Regulation by sex steroids and offspring stimuli Ann N Y Acad Sci 1997 807 126 145 9071347 10.1111/j.1749-6632.1997.tb51916.x

[B35] Champagne FA Weaver IC Diorio J Sharma S Meaney MJ Natural variations in maternal care are associated with estrogen receptor alpha expression and estrogen sensitivity in the medial preoptic area Endocrinology 2003 144 4720 4724 12959970 10.1210/en.2003-0564

[B36] Numan M Rosenblatt JS Komisaruk BR Medial preoptic area and onset of maternal behavior in the rat J Comp Physiol Psychol 1977 91 146 164 402400 10.1037/h0077304

[B37] Champagne FA Weaver IC Diorio J Dymov S Szyf M Meaney MJ Maternal care associated with methylation of the estrogen receptor-alpha1b promoter and estrogen receptor-alpha expression in the medial preoptic area of female offspring Endocrinology 2006 147 2909 2915 16513834 10.1210/en.2005-1119

[B38] Ogawa S Eng V Taylor J Lubahn DB Korach KS Pfaff DW Roles of estrogen receptor-alpha gene expression in reproduction-related behaviors in female mice Endocrinology 1998 139 5070 5081 9832446 10.1210/endo.139.12.6357

[B39] Stolzenberg DS Rissman EF Oestrogen-independent, experience-induced maternal behaviour in female mice J Neuroendocrinol 2011 23 345 354 21276101 10.1111/j.1365-2826.2011.02112.x PMC3064747

